# Association Between Food Insecurity and Diet Quality Among Early Care and Education Providers in the Pennsylvania Head Start Program

**DOI:** 10.5888/pcd18.200602

**Published:** 2021-06-17

**Authors:** Dania Mofleh, Nalini Ranjit, Ru-Jye Chuang, Jill N. Cox, Christine Anthony, Shreela V. Sharma

**Affiliations:** 1 Department of Epidemiology, Human Genetics and Environmental Sciences, Michael & Susan Dell Center for Healthy Living, The University of Texas Health Science Center at Houston School of Public Health, Houston, Texas; 2 Department of Epidemiology, Human Genetics and Environmental Sciences, Michael & Susan Dell Center for Healthy Living, The University of Texas Health Science Center at Houston School of Public Health, Austin, Texas; 3 Penn State Extension Better Kid Care, State College, Pennsylvania

## Abstract

**Introduction:**

Food insecurity affects dietary behaviors and diet quality in adults. This relationship is not widely studied among early care and education (ECE) providers, a unique population with important influences on children’s dietary habits. Our study’s objective was to explore how food insecurity affected diet quality and dietary behaviors among ECE providers.

**Methods:**

We used baseline data from a cluster-randomized controlled trial (January 2019−December 2020) on 216 ECE providers under the Pennsylvania Head Start Association. We used radar plots to graph scores for the Healthy Eating Index 2015 and the Alternative Healthy Eating Index (AHEI) 2010 and fitted a multivariate regression model for diet quality measures, adjusting for covariates.

**Results:**

Among the 216 participants, 31.5% were food insecure. ECE providers who were food insecure had a lower AHEI-2010 mean score (mean difference for food insecure vs food secure = −4.8; 95% CI, −7.8 to −1.7; *P* = .002). After adjusting for covariates, associations remained significant (mean difference = −3.9; 95% CI, −7.5 to −0.4; *P* = .03). Food insecure ECE providers were less likely to use nutrition labels (22.8% vs 39.1%; *P *= .046) and more likely to report cost as a perceived barrier to eating fruits and vegetables.

**Conclusion:**

We found a significant inverse association between food insecurity and the AHEI-2010 diet quality score among ECE providers after adjusting for covariates. More studies are needed to examine the effects of food insecurity on dietary behaviors of ECE providers and their response to nutrition education programs targeting their health.

**Summary**

**What is already known about this topic?**

High rates of food insecurity were reported among early care and education (ECE) providers. Little research has examined the association between food insecurity and diet quality behaviors among ECE providers.

**What is added by this report?**

Our study confirmed the high prevalence of food insecurity among ECE providers. Food insecure ECE providers were less likely to use nutrition labels and more likely to report cost as a perceived barrier to eating fruits and vegetables.

**What are the implications for public health practice?**

Our results can help inform intervention strategies to mitigate food insecurity and improve diet quality among ECE providers.

## Introduction

More than 2 million early care and education (ECE) providers, mostly women, provide care to over 10 million preschool-age children in the US (1). As adults who take care of children for a substantial part of the day, they model and cultivate healthy eating behaviors essential to children’s long-term health and behavior outcomes (2,3). Therefore, the health and well-being of ECE providers are essential to a child’s early learning and development success (2). However, ECE providers are susceptible to poor diet quality, sedentary lifestyle, stress, and economic worry (4) because they are more likely to live in poverty than, for example, K-12 teachers (5), earn low wages (national median wage = $24,230) (6), are often uninsured, and lack support and flexibility in their work environment (1).

Recent studies confirmed high rates of food insecurity among ECE providers (2,7). Food insecurity is defined as household-level economic hardship that limits a person’s ability to access an adequate amount of food (8). Although 10.5% of US households are food insecure (9), the prevalence is triple that among ECE providers, with estimates ranging from 34.5% to 42% (2,7). Moreover, studies showed that ECE providers, like other food insecure populations, have low nutrition knowledge (10,11), low fruit and vegetable consumption, and high intake of unhealthy foods (11,12), all of which increase their risk of chronic conditions, such as diabetes, hypertension, and hyperlipidemia (13,14). Food insecurity is linked to low diet quality in the general US population (15); however, little research has examined the association between ECE providers’ food insecurity and their diet quality and dietary behaviors.

## Methods

We used baseline data from the Create Healthy Futures study (16) to conduct a cross-sectional analysis to estimate the prevalence of food insecurity and examine the association between food insecurity and diet quality among ECE providers employed at Head Start programs in Pennsylvania. The Create Healthy Futures study is a cluster-randomized controlled trial evaluating a web-based intervention developed by Penn State Extension Better Kid Care (https://extension.psu.edu/programs/betterkidcare). Our sampling frame consisted of Center-based ECE programs in Pennsylvania, operating under the Pennsylvania Head Start Association, that offered year-round education to children aged 0 to 5 years. We estimated that 182 providers were needed from a minimum of 16 Head Start sites to detect significant differences of at least 0.5 standard deviation units in dietary outcomes, with 80% power. Eligibility criteria for ECE providers were 1) being employed at a participating ECE site at the time of recruitment, 2) the ability to read and speak English, 3) having a working email address, and 4) providing care for children aged 0 to 5 years in a classroom setting. We recruited a total of 12 ECE programs that comprised 39 sites to participate in our study. We invited 428 ECE providers working at these sites via email to participate in the study. Of these, a convenience sample of 256 providers agreed to participate (60% recruitment rate); 216 ECE providers completed the baseline survey for the Create Healthy Futures clinical trial from October 2019 through January 2020. We obtained informed consent electronically by email prior to accessing the surveys. The University of Texas Health Committee for Protection of Human Subjects institutional review board approved the study protocol and data collection.

We administered all surveys through Research Electronic Data Capture (RedCap) and Qualtrics (Qualtrics XM), both of which are HIPAA compliant web-based software. The baseline survey took approximately 30 to 45 minutes to complete. ECE providers who completed the baseline survey received a $25 gift card for a retail store.

### Measures

Food insecurity was self-reported by using a previously validated 2-item questionnaire, the Hunger Vital Sign (17), with response options of “never true,” “sometimes true,” or “often true” to the following statements: “Within the past 2 months I worried whether our food would run out before we got money to buy more” and “Within the past 2 months the food I bought just didn’t last and I didn’t have the money to get more” (17).

Sociodemographic measures collected were self-reported sex, race/ethnicity, age, educational level, work history, and income. By using self-reported height and weight, we computed participants’ body mass index (BMI) (weight in kg/height in m^2^) (18).

We assessed perceived concern about life necessities with the following questions (19): “In the past month, how much concern about life necessities like having a place to live, having enough to eat, or feeling like you are safe bothered you?” with 7-point response options ranging from 1, “never,” to 7, “always” (19). We assessed capacity to deal with problems with the following question: “How sure are you that you can deal with problems that come up in your life?” The 7-point Likert scale response options ranged from 1, “very unsure” to 7, “very sure” (19). These 2 questions were summed after collapsing each item’s responses into 3 categories and reverse coding the question measuring capacity to deal with life problems. The resultant measure, “coping ability with life problems,” was used as a proxy for participants’ socioeconomic status (range, 0-4), with a higher score indicating a lesser ability to cope with life problems (19). We used 1 question to measure perceived stress: “In the last month, how often have you felt nervous and stressed?” A 5-point scale of response options ranged from 1, “never,” to 5, “very often” (19).

### Diet quality measures

The primary dependent variables were 2 measures of diet quality, the Healthy Eating Index (HEI) 2015 (20), and the Alternative Healthy Eating Index (AHEI) 2010 (21), as assessed from the 2014 Block Food Frequency Questionnaire (22), a self-reported tool measuring food frequency intake from a list of 127 food and beverage items. Scoring methods for HEI-2015 and AHEI-2010 were previously validated (21,23,24). HEI-2015 consists of 13 components, each representing a major food group. Collectively, the components yield a maximum score of 100, and a higher score indicates a better alignment with the Dietary Guidelines for Americans (23). Nine components represent adequacy (foods needed for overall good health): total fruit, whole fruit, total vegetables, greens and beans, whole grains, dairy, total protein foods, seafood and plant proteins, and fatty acids. Four components represent moderation (foods that should be limited in a diet): refined grains, sodium, added sugars, and saturated fats (20). AHEI-2010 was developed by using evidence-based recommendations to incorporate additional components focusing on food group nutrients that predict risk for chronic diseases (21,25). AHEI-2010 consists of 11 components that produce a maximum score of 110. Although there are no distinct adequacy and moderation subgroups, 6 components are considered adequacy components: total vegetables, total fruit, whole grain, nuts and legumes, fish fatty acids, and polyunsaturated fatty acids. One component; alcohol, can be considered a moderation component, and 4 components are not favorable: sugary beverages (any beverage with sugar, natural or added), fruit juices, red and processed meat, and trans fat (26).

### Dietary habits

We used various previously validated items to measure dietary habits (22). We used a 2-item questionnaire to measure the frequency of fruit and vegetable consumption (22) (eg, “How many fruits eaten per day or week”) with response options on a 9-point scale ranging from 1, “rarely,” to 9, “4 or more per day.” We used a 2-item questionnaire to measure frequency of meals and snacks consumption (22) (ie, “How many meals per day?”) with response options ranging from 1 to 5 times per day. We measured perceived barriers to eating fruits and vegetables by using 4 items from the Family Life, Activity, Sun, Health, and Eating Study (27). For example, “I don't eat fruits and vegetables as much as I like to because they cost too much.” The research team members added a fifth item, “I don’t know how to cook vegetables,” to this study. Responses were scored on a 5-point Likert scale, from 0 (“strongly disagree”) to 4 (“strongly agree”). We computed a summative scale for the perceived barrier to eating fruits and vegetables ranging from 0 to 20 (Cronbach’s α = 0.73).

We used 5 items to measure nutrition knowledge (16) (eg, “About how much of your plate should be fruits and vegetables?”) with response options of “one-quarter,” “one half,” “three-quarters,” or “all of it”). Each question consisted of 4 answer choices, with only 1 correct response recoded as 1 for correct and 0 for incorrect answers. The final knowledge index score ranged from 0 to 5. We used a single item to assess the use of nutrition labels to evaluate a provider’s ability to navigate the food environment (16): “How often do you use the nutrition facts label on foods and beverages to make your grocery purchasing decisions,” with answer choices of “never,” “rarely,” “sometimes,” “often,” and “always.”

### Statistical analysis

We used the Student *t* test for continuous variables, and the Pearson χ^2^ or Fisher exact test for categorical variables to examine distributional differences in the dependent variables and covariates across food insecure and food secure groups by using a 2-tailed *P* value of < .05 as a threshold for significance. We compared diet quality among food secure and food insecure ECE providers for HEI-2015 and AHEI-2010 component scores.

Of the 216 ECE providers, 16 (7.4%) refused to provide income information, and 1 (0.5%) had missing information for meal patterns. We used a multivariable linear regression analysis as our main method to assess the association between diet quality and food insecurity status and to assess the association between food insecurity and dietary behaviors and diet-related psychosocial factors, including nutrition knowledge and perceived barriers to consumption of fruits and vegetables. Our final adjusted model included the following covariates: age, BMI, income, employment status (full-time vs part-time), coping ability with life problems, and work duration at the facility. All models relied on listwise deletion to handle missing data. Finally, we used a multivariable logistic regression analysis to assess the predicted probability of using nutrition labels to make grocery purchasing decisions by food insecurity status and Poisson regression to assess predicted counts for the frequency of fruit and vegetable consumption and the number of meals and snacks consumed per day. Significance was established at *P *< .05.

Because data were collected as part of a cluster-randomized clinical trial, we calculated the intraclass correlation coefficient (ICC) for ECE programs (ICC = 0.0075) and sites (ICC = 0). These small values suggested that observations were independent and that multilevel models were not required. We formally tested linearity assumptions of the 2 primary dependent variables, HEI-2015 and AHEI-2010. We also tested the homogeneity of variance. We conducted all analyses using STATA 15.0 statistical software (StataCorp LLC).

## Results

A total of 216 ECE providers completed the baseline survey (50.5% response rate). The prevalence of food insecurity was 31.5% among our sample of ECE providers in fall 2019 (Table 1). Participating ECE providers were predominantly women (97.7%), White (78.2%), and had a mean age of 41.1 (standard deviation [SD], 11.9 y). About 44% had some college education or less, 33% had a household income from all sources of less than or equal to $25,000, and about 28% had concerns about life necessities.

**Table 1:** Sociodemographic Characteristics, by Food Security Status, Early Care and Education Providers (N = 216), Pennsylvania Head Start Association, January 2019–December 2020^a^

Characteristic	Total, N = 216	Food Secure, n = 148 (68.5%)	Food Insecure, n = 68 (31.5%)	*P* Value^b^
**Age, mean (SD)**	41.1 (11.9)	42.5 (12.5)	37.8 (9.7)	.01
**Sex**				
Male	5 (2.3)	5 (3.4)	0	.33
Female	211 (97.7)	143 (96.6)	68 (100)	
**Race/ethnicity**				
White	169 (78.2)	120 (81.1)	49 (72.1)	.14
Non-White	46 (21.8)	28 (18.9)	19 (27.9)	
**Body mass index (weight in kg/height in m^2^) , mean (SD)**	30.1 (8.0)	29.0 (6.6)	32.4 (10.1)	.046
**Education**				
Some college education or less	95 (44.0)	60 (40.5)	35 (51.5)	.13
College degree	121 (56.0)	88 (59.5)	33 (48.5)	
**Current position**				
Teacher	115 (53.2)	82 (55.4)	33 (48.5)	.52
Assistant teacher	74 (34.3)	47 (31.8)	27 (39.7)	
Other	27 (12.5)	19 (12.8)	8 (11.8)	
**Program type** c				
Center-based Head Start	173 (80.1)	118 (79.7)	55 (80.9)	.84
Home-based Head Start	11 (5.1)	7 (4.7)	4 (5.9)	.74^d^
Preschool or public school Pre-K	37 (17.1)	24 (16.2)	13 (19.1)	.60
**Duration of work at the ECE facility, y**				
1–5	128 (59.2)	86 (58.1)	42 (61.8)	.04
6–10	36 (16.7)	20 (13.5)	16 (23.5)	
>10	52 (24.1)	42 (28.4)	10 (14.7)	
**Annual income from all sources** e				
≤25,000	66 (33.0)	37 (27.4)	29 (44.6)	.004
25,000–35,000	56 (28.0)	34 (25.2)	22 (33.9)	
35,000–50,000	32 (16.0)	28 (20.7)	4 (6.1)	
>50,000	46 (23.0)	36 (26.7)	10 (15.4)	
**Has concerns about life necessities**				
Never or rarely	155 (71.8)	131 (88.5)	24 (35.3)	<.001
Occasionally	35 (16.2)	12 (8.1)	23 (33.8)	
Frequently or always	26 (12.0)	5 (3.4)	21 (30.9)	
**Ability to deal with problems that come up in their life**				
Very unsure/a little unsure	28 (13.0)	17 (11.5)	11 (16.2)	.57
Neutral	23 (10.6)	17 (11.5)	6 (8.8)	
A little sure/very sure	165 (76.4)	114 (77.0)	51 (75.0)	
**In the last month, how often have you felt nervous and stressed**				
Never or almost never	16 (7.4)	12 (8.1)	4 (5.9)	.79
Sometimes or fairly often	144 (66.7)	99 (66.9)	45 (66.2)	
Very often	56 (25.9)	37 (25.0)	19 (27.9)	

Abbreviation: ECE, early childhood education.

a Values are number (percentage) unless otherwise indicated.

b *P* value calculated by using χ^2^ for categorical variables unless specified otherwise. Significant at *P* < .05.

c Category totals do not equal total sample size because of multiple value selections.

d Fisher Exact Test used to calculate *P* value.

e Data missing for 16 people who refused to answer.

Several socioeconomic measures differed significantly by food security status among ECE providers. Food insecure providers were younger (mean age, 37.8 y for food insecure vs mean 42.5 y for food secure, *P* = .01), had higher self-reported BMI (mean = 32.4 kg/m^2^ for food insecure vs 29.0 kg/m2 for food secure, *P* = .046), were less likely to have worked for more than 10 years at the ECE facility (14.7% for food insecure vs 28.4% for food secure, *P *= .04), and less likely to earn higher wages, defined as an annual income of $35,000 to $50,000 (6.1% for food insecure vs 20.7% for food secure, *P *= .004) (Table 1). A higher proportion of food insecure ECE providers reported having occasional or constant concerns about life necessities, such as having a place to live, feeling safe, and having enough to eat, compared with their food secure counterparts (64.7% vs 11.5%, *P* < .001).

We constructed radar plots to visualize the unadjusted differences in intakes of foods from multiple component food groups across food insecure and food secure ECE providers for diet quality measures of both HEI-2015 and AHEI-2010 (Figure). Overall, the median HEI-2015 score for ECE providers was less than for fatty acid ratio, sodium, and saturated fatty acids (Figure A). When stratified by food security status, compared with food secure ECE providers, food insecure ECE providers reported a median score of approximately 30% lower for seafood and plant proteins (*P* = .02), a 15% lower median score for whole fruits (*P* = .38), a 14% lower median score for total vegetables (*P* = .09), 13% lower scores for added sugars (*P* = .11), a 5% higher score for sodium (*P* = .35), and 14% higher scores for dairy (*P* = .29). The AHEI-2010 showed 50% or lower scores for total vegetables, total fruits, whole grain, fish fatty acids, sodium, and sugary beverages among all ECE providers (Figure B). The median score for fish fatty acid and sugary beverages was lower among food insecure ECE providers than among those who were food secure; (9%, *P* = .04) and (11%, *P* = .06), respectively. Overall, the AHEI-2010 scores were lower among those who are food insecure than among food secure ECE providers (mean, 49.1 [SD, 9.6] vs mean, 53.9 [SD, 1.0]; *P* = .002).

**Figure:**
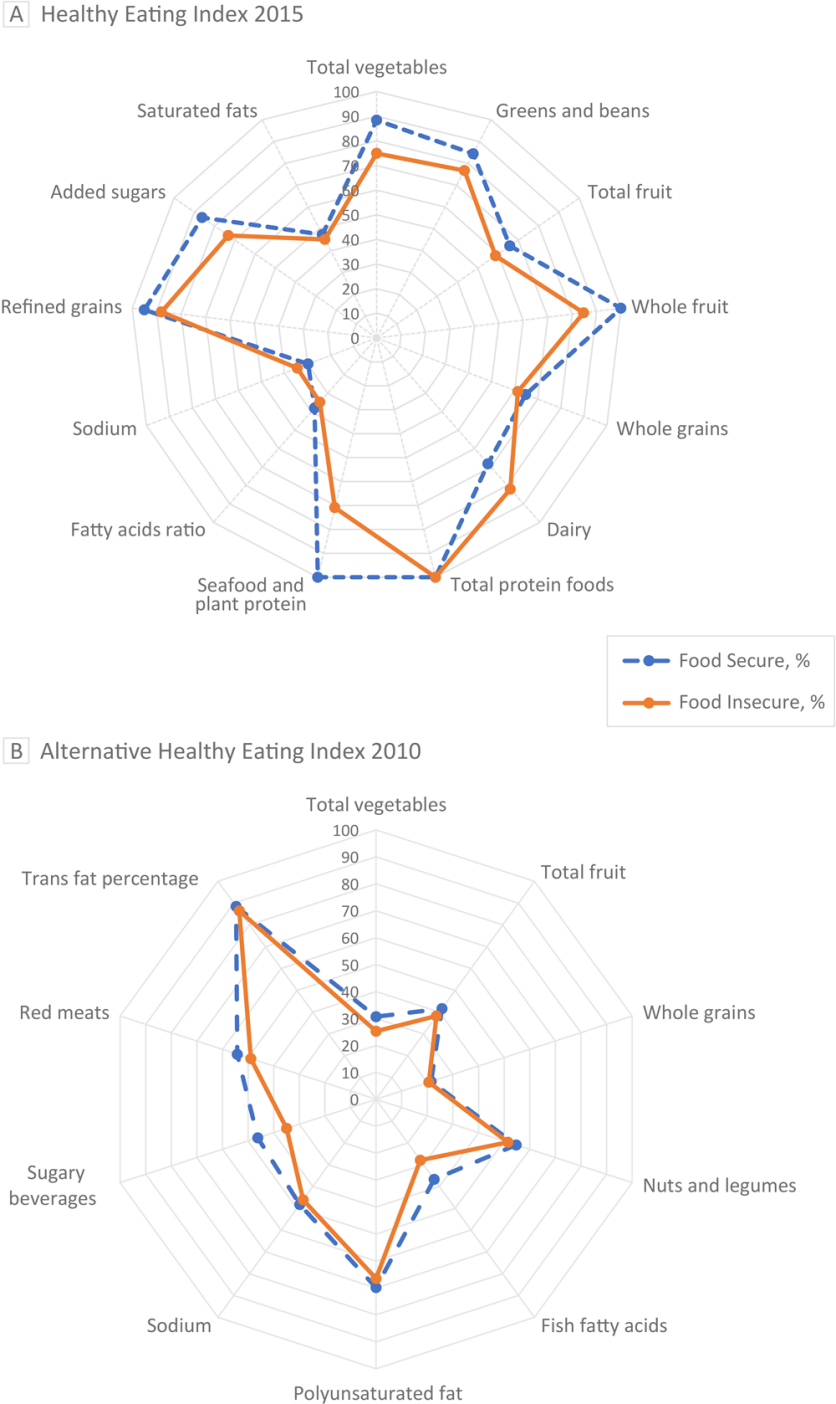
Radar plots of Healthy Eating Index (HEI) 2015 and Alternative Healthy Eating Index (AHEI) 2010 food components for both food secure and food insecure early childhood education providers. The radial axes represent median scores for food components graphed as percentages of each component’s total maximum score. The radar plots’ outer edges represent a maximum score of 100%, while the centers represent a minimum score of 0%. Plot A illustrates trends from HEI-2015. Total fruit represents all forms of fruit, including fruit juice; whole fruit represents all forms of fruit except fruit juice. Plot B illustrates trends from AHEI-2010. The median score for food secure was 53.1. For food insecure, the median score was 49.4. A higher score indicates a higher diet quality. Sugary beverages are any beverage with natural or added sugar.

**Table:** 

Plot Components	Overall, %	Food Secure, %	Food Insecure, %
**A. HEI-2015 **			
Median	61.4	62.2	59.9
Total vegetables	80.8	88.4	74.9
Greens and beans	81.5	84.5	76.8
Total fruit	65.3	65.8	58.7
Whole fruit	100.0	100.0	84.7
Whole grains	32.2	64.8	61.3
Dairy	69.9	68.2	81.9
Total protein foods	100.0	100.0	100.0
Seafood and plant protein	94.8	100.0	70.9
Fatty acids ratio	37.1	38.0	34.8
Sodium	31.3	29.8	34.4
Refined grains	93.0	95.0	88.0
Added sugars	80.9	86.1	73.2
Saturated fats	46.4	47.4	45.2
**B. AHEI-2010**			
Median overall	47.8	53.1	49.4
Total vegetables	29	30.7	25.3
Total fruit	40.5	41.6	38.2
Whole grains	21.2	21.5	20.6
Nuts and legumes	53.7	54.7	51.5
Fish fatty acids	33.9	36.7	27.9
Polyunsaturated fat	68.8	69.9	66.5
Sodium	47.6	48.3	46.1
Sugary beverages	42.6	46.2	35
Red meat	52.6	54.2	48.9
Trans fat	87.8	88.5	86.5

For AHEI-2010 diet quality measures, regression analysis results examining the association between food insecurity and diet quality measures showed a significant inverse association with food insecurity (Table 2). The unadjusted model showed that ECEs who were food insecure had significantly lower AHEI-2010 scores than those who were food secure (mean difference, −4.8; 95% CI, −7.8 to −1.7; *P* = .002). These associations remained significant after controlling for covariates (mean difference, −3.9; 95% CI, −7.5 to −0.4; *P* = .03). We also saw an inverse association between food insecurity and HEI-2015, but it was not significant.

**Table 2:** Mean Difference in Diet Quality Scores, by Food Security Status, Using Unadjusted and Adjusted Models, Early Care and Education Providers (N = 216), Pennsylvania Head Start Association, January 2019–December 2020

Model	Unadjusted Model				Adjusted Model^a^			
	Food Secure	Food Insecure	Difference Across Groups		Food Secure	Food Insecure	Difference Across Groups	
	Mean (SE)	Mean (SE)	Mean Difference **b** (95% CI)	*P* Value^c^	Mean **d** (SE)	Mean **d** (SE)	Mean Difference **b** (95% CI)	*P* Value^c^
**Healthy Eating Index 2015**	62.2 (0.8)	60.2 (1.1)	−2.0 (−4.7 to 0.7)	.152	62.2 (0.8)	60.4 (1.3)	−1.8 (−4.9 to 1.4)	.27
**Alternative Healthy Eating Index 2010**	53.9 (0.9)	49.1 (1.3)	−4.8 (−7.8 to −1.7)	.002	53.6 (0.9)	49.7 (1.4)	−3.9 (−7.5 to −0.4)	.03

Abbreviations: SE, Standard Error.

a Adjusted models controlled for age, body mass index (weight in kg/height in m^2^), income, employment status, duration of work at facility, and ability to cope with life problems.

b Mean difference represents the difference in means between food secure and food insecure ECE providers.

c Significant at *P* < .05.

d The predicted adjusted mean represents the average mean for Alternative Healthy Eating Index 2015 or Alternative Healthy Eating Index 2010 for each group (food secure vs food insecure) obtained from the adjusted models after controlling for age, body mass index (weight in kg/height in m^2^), income, employment status, duration of work at facility, and ability to cope with life problems.

Food insecure ECE providers reported consuming fewer meals per day than their food secure counterparts (adjusted predicted counts, 2.6 vs 2.9 meals per day; *P* = .03) (Table 3). Furthermore, the frequency of use of nutrition labels to make grocery purchasing decisions was significantly lower among those who were food insecure than among their food secure counterparts (22.8% vs 39.1%; *P* = .046). We also assessed the relationship between food insecurity and perceived barriers to eating fruits and vegetables. We found that food insecure providers were more likely to report cost of food as being a perceived barrier to eating fruits and vegetables than their food secure counterparts (37.2% vs 23.3%; *P* = .03) after adjusting for age, BMI, income, employment status, coping ability with life problems, and work duration at the Head Start facility.

**Table 3:** Differences in Dietary Behaviors and Perceptions Across Food-Secure and Food-Insecure Early Care and Education Providers (N = 216), Pennsylvania Head Start Association, January 2019–December 2020^a^

Modifiable Risk Factors	Unadjusted			Adjusted^b^		
	Food Secure, Mean (SE)	Food Insecure,Mean (SE)	*P* Value^c^	Food Secure, Mean (SE)	Food Insecure, Mean (SE)	*P* Value^c^
**Meal patterns^d^^,^^e^**						
Vegetables eaten per day, no. of servings	1.2 (0.1)	1.0 (0.1)	.12	1.2 (0.1)	1.1 (0.1)	.61
Fruits eaten per day, no, of servings	1.0 (0.1)	0.9 (0.1)	.33	1.0 (0.1)	0.9 (0.1)	.46
Number of meals per day	2.9 (0.1)	2.7 (0.1)	.02	2.9 (0.1)	2.6 (0.1)	.03
Number of snacks per day	1.9 (0.1)	2.1 (0.1)	.12	1.9 (0.1)	2.0 (0.1)	.78
**Perceived barriers to eating fruits and vegetables**^f^	6.1 (0.3)	7.3 (0.5)	.04	6.2 (0.4)	6.8 (0.6)	.37
**Nutrition knowledge index**^g^	3.2 (0.1)	3.1 (0.1)	.58	3.2 (0.1)	3.2 (0.1)	.96
**Use nutrition labels^h^, % (SE)**						
Never to sometimes	60.2 (0.0)	78.0 (0.1)	.01	60.9 (0)	77.2 (0.1)	.046
Often to always	39.8 (0.04)	22.0 (0.1)		39.1 (0)	22.8 (0.1)	

Abbreviations: SE, standard error.

a Multivariable linear regression analysis reported means unless specified otherwise.

b Adjusted means were controlled for age, body mass index (weight in kg/height in m^2^), income, employment status, duration of work at facility, and coping abilities with life problems.

c Significant at *P* < .05.

d Predicted counts, obtained from a Poisson regression analysis.

e Data missing for 1 observation.

f Scale is a 5-point Likert scale (strongly disagree = 0 and strongly agree = 4.) Scores were converted to a 20-point scale for analysis. A higher score indicated a higher perceived barrier of healthy eating.

g The Nutrition Knowledge Index is a 5-item subscale. Each item is scored from 0 (least knowledge) to 1 (greatest knowledge); the sum was used for analysis.

h Predicted probabilities, reported as percentages obtained from a logistic multivariable regression analysis.

## Discussion

Our study demonstrated that food insecure ECE providers had lower diet quality and were consuming significantly fewer meals per day than their food secure counterparts. The prevalence of food insecurity in our sample, 31.5%, was high and higher than the national average, although it was consistent with the prevalence of food insecurity among low-income households (9). These rates of food insecurity are comparable with a recent study examining 307 ECE providers, which found that 34.5% were food insecure (7). The low national median wages for ECE providers of $24,230 (6) coupled with the high prevalence of food insecurity and poor diet quality seen in our population warrants immediate attention to the ECE environment and increased support to ECE providers in order to address their basic needs.

Overall HEI-2015 diet quality scores for ECE providers in our study were comparable to the national average of 58.4; however, the overall AHEI-2010 scores in our study population were higher than the national average of 41.8 (28), possibly because of differences in sex, socioeconomic status, and age distribution. In our study, food insecure ECE providers had HEI-2015 scores comparable to food secure providers; however, for AHEI-2010, the scores were significantly lower among those who were food insecure. These results align with those from a study that used National Health and Nutrition Examination Survey data that reported significantly lower overall scores in AHEI-2010 and a previous version of HEI-2015 dietary measures among food insecure adults compared with food secure adults in the US (15). Furthermore, food insecure ECE providers reported lower scores for fish fatty acids and sugary beverages per AHEI-2010. AHEI-2010 is designed to capture additional nutrition information on diet quality affecting preventable chronic diseases (24). Literature shows an association between low AHEI-2010 scores and increased risk for type 2 diabetes (25) and increased mortality rates for cardiovascular disease and cancer (29). These findings, along with those from our study, suggest that food insecurity among ECE providers could potentially predispose them to higher risks of chronic diseases in later life because of low diet quality (21,24,30); these relationships could be explored in future research. Our results can help inform intervention strategies to mitigate food insecurity and improve diet quality among ECE providers (15).

Our study also showed that food insecure ECE providers were less likely to read food labels often or always than food secure providers. This finding could be due to purchases being driven primarily by cost rather than the nutrition content of the foods. These results are consistent with those of previous studies of low-income households that report lower use of nutrition labels to navigate the food environment (31). Furthermore, our results showed that food insecure ECE providers were more likely to perceive barriers to eating fruits and vegetables than food secure providers, specifically barriers related to cost. Nutrition knowledge did not differ between the 2 groups. Programs targeting ECE providers’ healthy eating need to address environmental factors to reduce perceived barriers to eating fruits and vegetables (eg, enrollment of those eligible in the Supplemental Nutrition Assistance Program and the Special Supplemental Nutrition Program for Women, Infants, and Children) and provide skill-based nutrition education to improve food preparation, food budgeting, and use of nutrition labels to guide grocery shopping.

Head Start programs outline several domains that emphasize child health outcomes, including healthy nutrition. ECE providers, such as those in our study, are in a unique position to implement nutrition education and act as role models for healthy eating for children in their care (3); they can play critical roles in the success of interventions targeting childhood obesity (11). Our study’s results underscore the need to provide support to the Head Start ECE providers community to improve their own dietary behaviors so that they can effectively implement health education programs for children in their care. Furthermore, given the recent COVID-19 pandemic and its related financial crisis, which have increased food insecurity nationwide, our results demonstrate a call for further research to assess the pandemic’s impact on ECE providers who are among frontline workers (32).

Our study's strengths include the provisional insight it provides in assessing ECE providers’ nutrition needs. We calculated food insecurity by using validated measures and a coding scheme with high specificity (17). We used reliable measures to estimate dietary intake (22) and assessed a variety of dietary behavior indicators. Our study also had limitations. It may not adequately represent ECE providers across the US because the study sample was conducted in Pennsylvania only, limiting the study’s generalizability. Moreover, a selection bias may have been introduced because we used a convenience sample, and participation was voluntary; no information was available on the 40% of ECE providers who chose not to respond to the survey. We did not collect information about whether ECE providers were their household’s primary shopper and thus could not apply such information to our analysis. Potential issues also existed with the measures. Self-reported dietary intake measures are subject to social desirability bias; however, we used the validated Block Food Frequency Questionnaire. Coping ability with life problems, knowledge index, and navigating the food environment measures were not previously validated, although they demonstrated face validity. Finally, although the difference in AHEI-2010 mean scores across food secure and food insecure populations were significant, those differences were small and likely not meaningful in relation to risk for chronic disease. Nevertheless, the persistence of significance after adjustment suggests that this finding is robust.

Our study confirms a high prevalence of food insecurity among ECE providers and demonstrates that food insecurity is associated with lower diet quality, less frequent use of nutrition labels, and higher perceived barriers to consuming fruits and vegetables related to cost among food insecure providers than their food secure counterparts. These results warrant further investigation to inform the development of strategies mitigating food insecurity and promoting healthy eating behaviors in this ECE provider population.
